# EEG microstate features according to performance on a mental arithmetic task

**DOI:** 10.1038/s41598-020-79423-7

**Published:** 2021-01-11

**Authors:** Kyungwon Kim, Nguyen Thanh Duc, Min Choi, Boreom Lee

**Affiliations:** Department of Biomedical Science and Engineering (BMSE), Institute Integrated Technology (IIT), Gwangju Institute of Science and Technology (GIST), 123 Cheomdan-gwagiro, Buk-gu, Gwangju, 61005 South Korea

**Keywords:** Cognitive neuroscience, Imaging

## Abstract

In this study, we hypothesized that task performance could be evaluated applying EEG microstate to mental arithmetic task. This pilot study also aimed at evaluating the efficacy of microstates as novel features to discriminate task performance. Thirty-six subjects were divided into good and poor performers, depending on how well they performed the task. Microstate features were derived from EEG recordings during resting and task states. In the good performers, there was a decrease in type C and an increase in type D features during the task compared to the resting state. Mean duration and occurrence decreased and increased, respectively. In the poor performers, occurrence of type D feature, mean duration and occurrence showed greater changes. We investigated whether microstate features were suitable for task performance classification and eleven features including four archetypes were selected by recursive feature elimination (RFE). The model that implemented them showed the highest classification performance for differentiating between groups. Our pilot findings showed that the highest mean Area Under Curve (AUC) was 0.831. This study is the first to apply EEG microstate features to specific cognitive tasks in healthy subjects, suggesting that EEG microstate features can reflect task achievement.

## Introduction

Human cognition is based on neural activity. Brain networks ranging from individual neurons to large-scale systems are known to be the basis for cognition and behaviour^1^. Several brain functions are assumed to be supported by a specific area composed of complex organizations of neurons, resulting in so-called functional localization. Higher-order brain functions seem more likely to require complex patterns of activation across multiple brain areas rather than segregated modular function^2^. Cognitive functions such as working memory, attention, and planning are difficult to localize to a discrete area. Depending on the cognitive task, network analysis of functional magnetic resonance imaging (fMRI) data shows dynamic reorganization, including segregation and integration states^3,4^. In this context, an analysis of the dynamic network is essential to investigate the behaviour associated with complex cognitive function.

Many previous studies have investigated the efficiency of cognitive functions by network analysis using fMRI. For example, working memory, including the function of maintaining relevant information and allowing mental manipulation of this information^5,6^ has been widely studied in task performance measurements as a fundamental component involved in most higher-order brain functions. In language processing tasks, individuals with higher working memory capacity show increased activation of the anterior cingulate cortex compared to those with lower capacity^7^. Working memory performance engages top-down modulatory functions, which can be assessed by the functional connectivity of prefrontal and parietal control regions^8^. Furthermore, Andre et al. reported that cognitive function maturation following brain development requires changes in working memory networks^9^. They reported several activations in the localised cortical areas including the increased and decreased activation of specific networks, suggesting that the development of higher-order functions can also be interpreted by network analysis. In prospective studies, improvement in cognitive performance was associated with changes in functional organisation^10^. In summary, many studies have shown that task performance is reflected in functional networks; therefore, it may be possible to evaluate cognitive task performance using an objective method of network analysis.

However, previous studies using fMRI have encountered a fundamental limitation in higher-order function research. fMRI does not measure neural activity directly, but rather measures indirect physiological changes such as neurovascular coupling and hemodynamic responses^11^. Because fluctuations in blood-oxygenation-level-dependent (BOLD) signals are relatively slow compared to neural activity, the range of functional connectivity that can be accurately measured with fMRI is limited^2^. Therefore, a method to supplement the weaknesses of the information provided by fMRI may be helpful, and it is needed to study higher-order functions using high temporal resolution.

Electroencephalography (EEG) allows the measurement of electrical activity on a millisecond scale that is commensurate with the rate of neuronal firing^2^. Based on the rationale that different topographic representations reflect different functional states^12^, analysis of the spontaneous EEG in short time segments revealed that there are several topographic orientations that switch rapidly^13^. This method of analysis is referred to as microstate analysis, wherein each successive signal represents a microstate^13,14^. Microstates remain quasi-stable for 60–120 ms, which is important for investigating brain dynamics^15^. Using a clustering algorithm, microstates can be classified into several groups based on topological similarity^16^.

Several studies have supported that cognitive manipulation of EEG microstates is possible^17–21^. It was reported that stress response was estimated using EEG microstate sequences in psychosocial stress paradigm^17^. In the semantic memory task, pre-stimulus EEG microstates showed correlation with known electrophysiological markers, which means they can reflect cortical activation^18^. When performing a word-pair memory task, it was observed that the network dynamics of the memory consolidation process changed EEG microstates^19^. There are also studies that have revealed the association between EEG microstates and the changes in cortical activity induced by brain stimulation. EEG microstate showed a correlation with inhibition applied to a specific region in resting state^20^ and with stimulation applied to a region mediating perceptual or memory task^21^. Since EEG microstates are associated with cognitive manipulation, it may be possible to evaluate cognitive functions using them.

However, most of these studies are based on a data-driven approach, they have several critical drawbacks. First, clustering algorithms do not result in a specific optimal numbers of clusters that is consistent across analyses of independent datasets^22,23^. Second, it is difficult to interpret the results in terms of neurosciences because the topological maps of the computed microstates are not template. If we can use the archetype microstates whose functional significance is well defined in each archetype microstate, which help to precise interpretation of the results regarding to neuroscience. Third, the optimal number of clustering extracted using K-means algorithms requires appropriate choices of clusters at initialisation steps. However, choosing a proper number of cluster can be difficult particularly in case the data is dynamic and prior knowledge is unknown. In addition, K-means method also starts with random choices of cluster centroids and therefore it may yield different clustering results on different iterations, which leads to inconsistent results. Archetype microstates, the same topographic presentation based on a lot of prior knowledge, will enable efficient and high interpretability to reveal underlying processes of mental activity.

A method of setting four prototypical microstates (types A, B, C, and D) in k-means clustering proposed by Lehmann et al. has been applied in many studies that have led to a better understanding of neurobiological bases^12,24^. This method has made it possible to show differences in microstates across groups and cognitive states by using the same archetype microstate features (i.e.; duration, occurrence, and coverage). The results of such analyses may have high interpretability because the functional significance of the four archetype microstates have been reported in several studies^25–27^. Schiller et al. described the network dynamics of oxytocin from the perspective that the duration and occurrence of microstate represents state of network^28^. The functions of microstates type A, B, C, and D are associated with fMRI resting state networks of auditory, visual, default mode, and dorsal attention, respectively^27^. Many studies have applied the four archetype microstate features to various psychiatric conditions^22,29–33^ general medical conditions^28,34^, and cognitive tasks^27,35^. Especially in schizophrenia, where impairments in cognitive function are well established, changes in microstate are reported according to various hypotheses^12,30,36–39^. In summary, four prototypical microstates are features that reveal their function and interpretation, making them suitable for developing models that can be applied to many diseases and tasks.

To date, microstate analysis has not been applied to assessment of task performance in higher-order brain functions that require changes in the brain network. Since complex cognitive operations demand changes in the brain network, cognitive functions may be evaluated with microstate features that can reflect brain networks. Recently, Seitzman et al.^27^ described changes in microstate features during a mental arithmetic task based on studies that revealed the association between microstate features and specific brain systems^25,26^. Type B features changed depending on whether visual input was present, while there was no change in type A features. Task-positive type D features increased and task-negative type C features decreased during a serial subtraction task. Several studies have reported similar results in type D features, in mean duration and mean occurrence^24,37,40^. As mean duration and mean occurrence reflect temporal dynamics and usage, respectively, differences in task achievement may be reflected in those in the global network. Because proper execution of higher-order function is associated with changes in characteristic microstate features, it may be possible to evaluate task performance using EEG microstate features.

Although there have been microstate feature-based studies on many diseases and cognitive tasks, there are few studies on classification using these microstate features. For example, Negar et al., investigated EEG microstate features for classification of epilepsy and PNES, and their findings showed that when the coverage feature of the EEG microstate analysis is calculated in beta-band, the classification showed fairly high accuracy and precision^41^. In addition, Kiran Raj et al., used machine learning techniques to explore if abnormalities in EEG microstates can identify patients with temporal lobe epilepsy^34^. It is necessary to examine the efficacy of microstates as a meaningful and novel feature, which can be used in combination of a classifier for predictions of test individuals. In other words, we can develop a trained classifier based on the classification analysis for taking microstate data from new yet-to-be-seen individuals as inputs and provide the predictions whether they are good or poor performers. In addition, we can develop a comparative protocol where we compare the classification performances of our microstate analysis features using feature selection method. However, the efficiency of microstate analysis features is still ambiguous for classification, and extensive investigation on this topic is of importance. In general, it might be true that if there are great differences between groups, the classification accuracies might be high. However, this is not always the case; the accuracy of classification must be a compromise between the novel extracted features from the raw dataset and a classifier. In some cases, even there are small differences between groups, the classification accuracies can be high if we have a well-designed classifier. In summary, the efficiency of microstates as novel features for classification needs to be investigated because many studies have not been performed despite the uncertainty.

In this study, we investigated the differences between known microstate features of good performers and poor performers during a mental arithmetic task. We hypothesised that microstate features would reflect performance on the mental arithmetic task. Among them, some features may be more useful, such as type D features (associated with dorsal attention), mean duration (associated with temporal stability), and mean occurrence (associated with usage). First, we obtained microstate features from EEG recordings during resting state and task state in good performers and poor performers. Next, we compared microstate features within groups and between groups during the task state. We also examined the possibility of using microstate characteristics as novel features for discriminations of cognitive tasks. Finally, we calculated the classification accuracies of microstate features obtained using RFE with machine-learning-based multivariate analysis in good and poor performers.

## Results

### Microstate analysis

**Figure 1:**
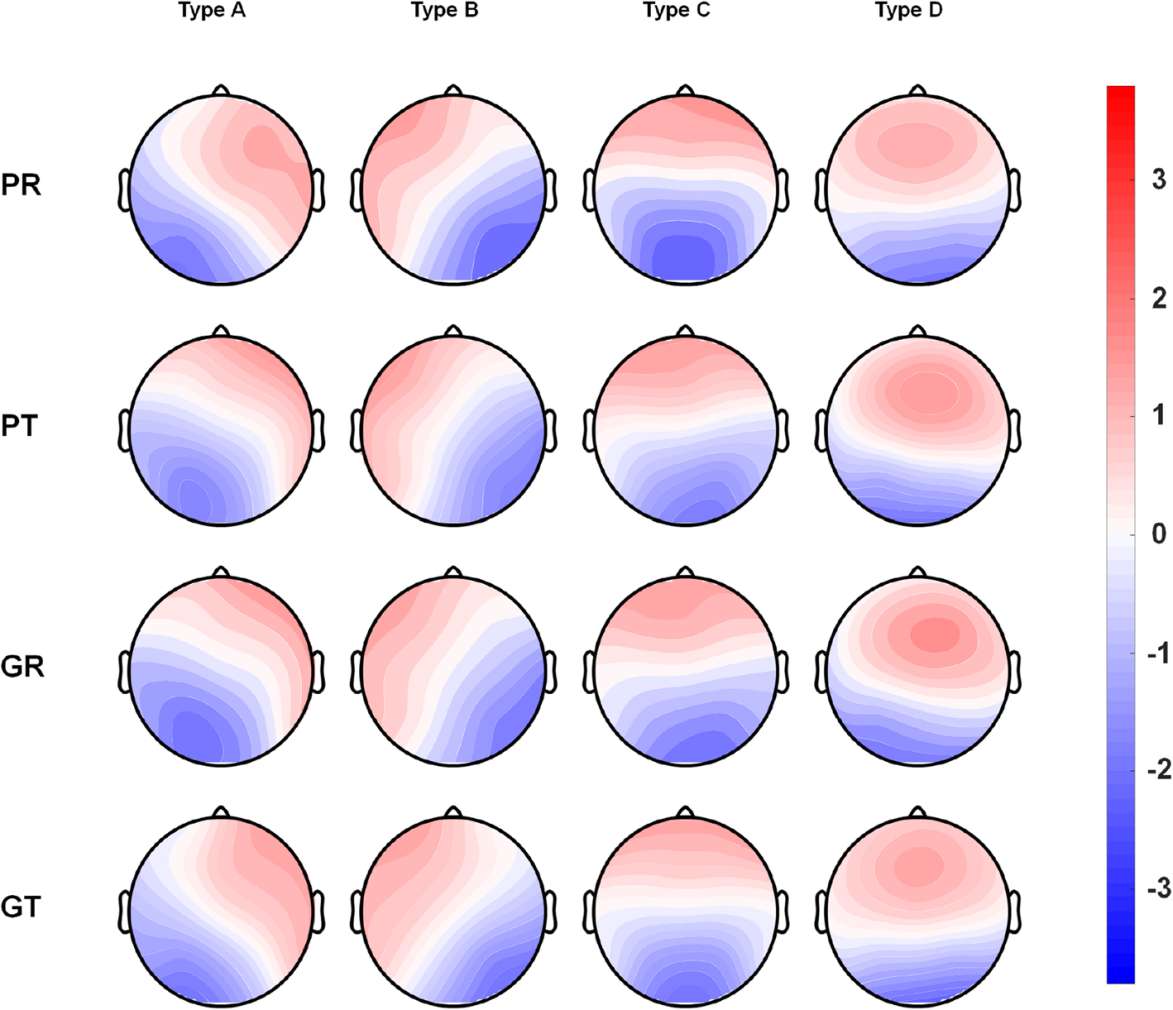
Four microstates of EEG recordings from resting and task states were obtained in good performers and poor performers. *PR* poor performers in the resting state, *PT* poor performers in task state, *GR* good performers in resting state, *GT* good performers in task state.

Normalised microstate scalp maps are shown in Fig. 1, exhibiting four states. In accordance with the labels applied in previous studies^12,24^, unilateral frontal orientations were labelled type A and B, anterior–posterior orientation was labelled type C, and the somewhat dominant orientation in the occipital area was labelled type D. Microstate analysis was performed by clustering based on the four archetype microstates.

The results presented in Table 1 show the comparison of the four states (PR: poor performers in the resting state, PT: poor performers in task state, GR: good performers in resting state, GT: good performers in task state) using 18 microstate features obtained from microstate analysis. From the two comparisons (PR vs. PT and GR vs. GT), differences in microstate features between good performers and poor performers are revealed in the task state and the resting state. In good performers, the mean duration decreased (p < 0.001) and the mean occurrence increased (p < 0.001). No type A features differed between the task and resting states. In type B features, only the duration was reduced (p = 0.002). The duration (p < 0.001) and coverage (p < 0.001) of type C microstates decreased between the task and resting states. For type D features, duration (p < 0.001), occurrence (p < 0.001), and coverage (p < 0.001) all increased. On the other hand, in the poor performer group, there was no difference in duration, unlike occurrence (p < 0.001) and coverage (p < 0.001) in type D feature and occurrence increased (p = 0.014) in type C. For types A and B, similar to good performers, only the duration of B decreased (p < 0.001). The mean duration also decreased (p < 0.001) and the mean occurrence increased (p < 0.001) between the task and resting states. In the two comparisons, all global field potential (GFP) features were reduced. In summary, type D features increased, type C features decreased, and type A and B features did not differ or not affect coverage in good performers during the mental arithmetic task. For poor performers, the occurrence of type C features increased, while the duration of type D features did not differ.

**Table 1:** Eighteen microstate features were obtained for the resting and task state EEG recordings in good performers and poor performers.

	PR	PT	GR	GT	Adjusted p-value and z-value after post-hoc test					
					PR vs PT		GR vs GT		PT vs GT	
**Duration (ms)**					z	p	z	p	z	p
A	63.6 (± 22.1)	61.4 (± 17.8)	61.5 (± 19.2)	60.4 (± 17.1)	1.845	0.195	1.701	0.195	0.730	0.466
B	68.3 (± 24.6)	62.0 (± 16.4)	66.9 (± 23.5)	64.3 (± 20.1)	4.404	< 0.001*	3.369	0.002*	− 1.964	0.099
C	94.2 (± 45.1)	86.8 (± 41.2)	99.2 (± 51.0)	92.3 (± 44.5)	4.679	< 0.001*	5.282	< 0.001*	− 2.120	0.068
D	72.4 (± 24.9)	68.7 (± 19.3)	71.5 (± 25.6)	76.2 (± 24.6)	− 1.103	0.540	− 3.804	0.001*	− 0.551	0.582
Mean duration	78.0 (± 18.2)	72.5 (± 14.9)	79.2 (± 20.3)	77.1 (± 16.8)	4.938	**< 0.001**	4.041	< 0.001*	− 2.702	0.014*
**Occurrence (Hz)**										
A	2.62 (± 1.20)	2.70 (± 1.05)	2.55 (± 1.21)	2.44 (± 1.17)	1.802	0.215	− 0.804	0.422	2.064	0.156
B	3.00 (± 1.16)	3.21 (± 1.15)	2.98 (± 1.19)	2.98 (± 1.09)	− 0.132	> 0.999	− 0.948	**> 0.999**	− 0.283	2.332
C	4.16 (± 1.13)	4.47 (± 1.04)	4.17 (± 1.14)	4.15 (± 1.11)	− 2.914	0.014*	− 0.967	0.667	1.864	0.187
D	3.62 (± 1.25)	3.88 (± 1.28)	3.58 (± 1.29)	3.91 (± 1.20)	− 5.802	< 0.001*	− 5.651	< 0.001*	2.906	**0.007**
Mean occurrence	13.38 (± 2.59)	14.25 (± 2.38)	13.29 (± 2.75)	13.48 (± 2.46)	− 4.938	< 0.001*	− 3.976	< 0.001*	2.702	0.014*
**Coverage (%)**										
A	16.5 (± 8.4)	16.3 (± 7.3)	15.6 (± 8.5)	14.7 (± 7.8)	− 0.184	> 0.999	0.540	**> 0.999**	2.385	0.051
B	20.5 (± 10.4)	19.9 (± 8.0)	19.8 (± 9.8)	19.3 (± 9.2)	2.335	0.078	0.916	0.593	− 1.117	0.792
C	37.3 (± 13.5)	37.0 (± 12.3)	39.2 (± 15.2)	36.6 (± 13.5)	2.501	0.025*	5.015	< 0.001*	− 0.602	0.547
D	25.8 (± 10.7)	26.7 (± 11.0)	25.3 (± 11.1)	29.5 (± 11.3)	− 5.432	< 0.001*	− 7.501	< 0.001*	1.303	0.327
**Mean GFP (µV)**										
A	5.51 (± 1.37)	4.86 (± 1.34)	5.29 (± 1.72)	4.90 (± 1.44)	3.936	< 0.001*	3.378	< 0.001*	1.804	0.071
B	5.68 (± 1.60)	4.90 (± 1.32)	5.43 (± 1.74)	5.05 (± 1.64)	4.796	< 0.001*	3.793	< 0.001*	1.076	0.282
C	6.12 (± 1.56)	5.31 (± 1.33)	5.94 (± 1.97)	5.46 (± 1.77)	4.464	< 0.001*	4.497	< 0.001*	2.348	0.019*
D	5.98 (± 1.51)	5.20 (± 1.31)	5.73 (± 1.85)	5.40 (± 1.59)	3.581	0.001*	2.430	0.030*	1.920	0.055

Statistical analyses were performed using Mann–Whitney test. After Bonferroni–Holm correction for multiple comparisons, significant differences are indicated in asterisk and bold.

*PR* poor performers in the resting state, *PT* poor performers in task state, *GR* good performers in resting state, *GT* good performers in task state, *GFP* global field potential, mean (± standard deviation).

A comparison of PT and GT reflects whether the difference in task performance can be reflected in the EEG microstate feature (Table 1). Compared with good performers, the occurrence of type D features (p = 0.007) decreased in poor performers; the latter showed reduced mean duration (p = 0.014) and increased mean occurrence (p = 0.014). Type A, B, and C features did not differ between the two groups. In summary, type D features increased, temporal stability increased as mean duration increased and mean occurrence decreased during the mental arithmetic task in good performers.

**Figure 2:**
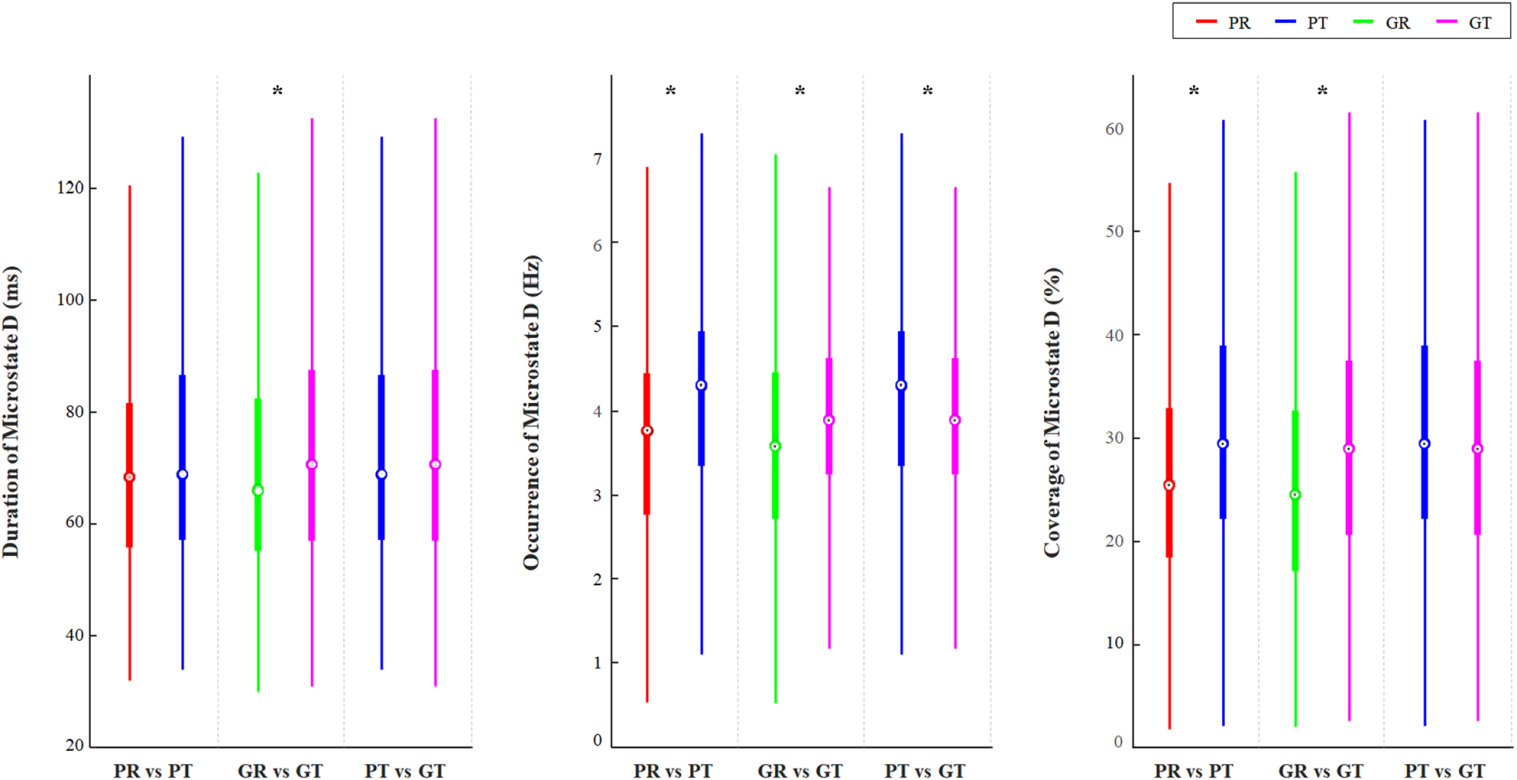
Changes in type D microstate features depending on task state and performance. Boxplot represents first quantile, third quantile, and the median. Group differences were tested with Mann–Whitney test. After Bonferroni–Holm correction for multiple comparisons, a significant difference is marked by an asterisk. *PR* poor performers in the resting state, *PT* poor performers in task state, *GR* good performers in resting state, *GT* good performers in task state.

**Figure 3:**
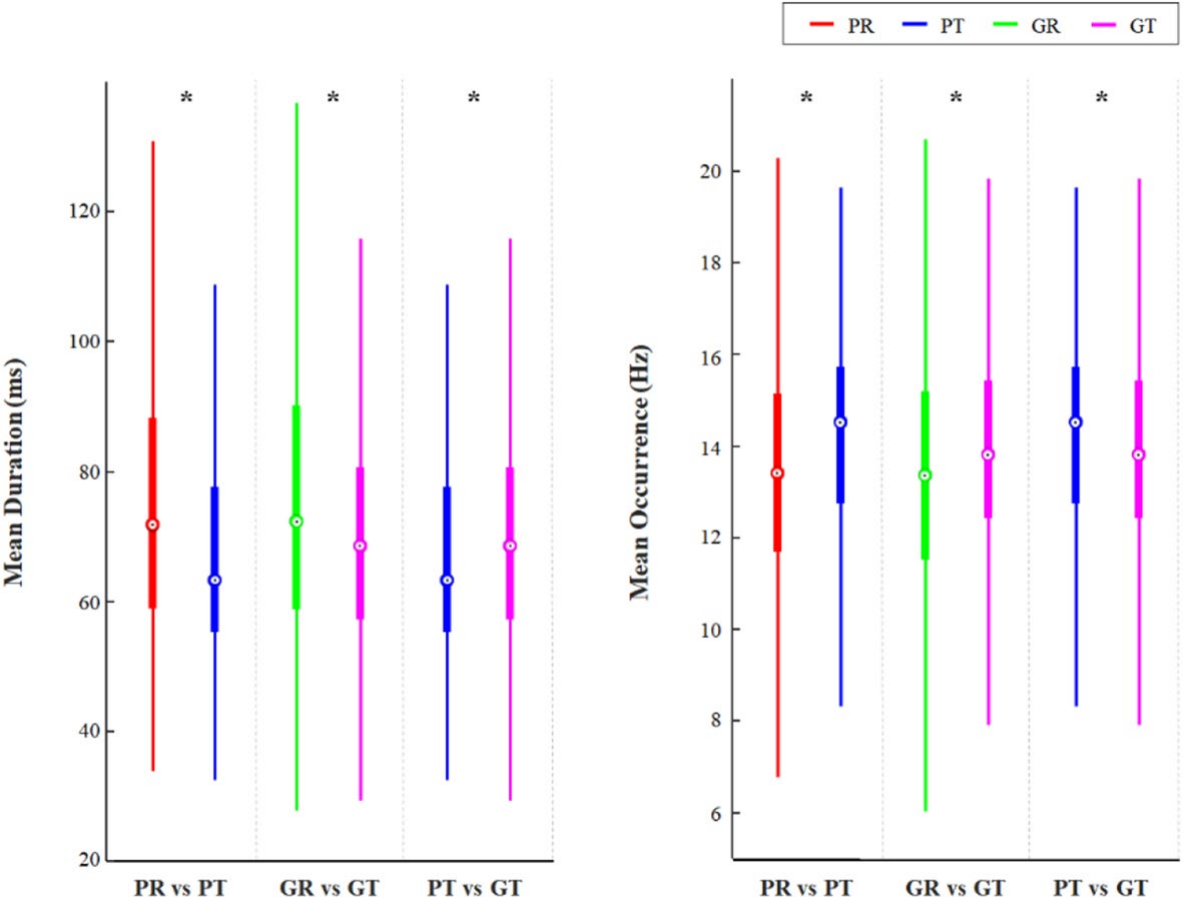
Changes in mean duration and mean occurrence depending on task state and performance. Boxplot represents first quantile, third quantile, and the median. Group differences were tested with Mann–Whitney test. After Bonferroni–Holm correction for multiple comparisons, a significant difference is marked by an asterisk. *PR* poor performers in the resting state, *PT* poor performers in task state, *GR* good performers in resting state, *GT* good performers in task state.

In particular, type D features, mean duration, and mean occurrence were investigated whether they exhibit the characteristics of four states. The change in type D features caused by the task is displayed in Fig. 2. For good performers (Fig. 2; middle columns), coverage increased with increasing duration and occurrence. On the other hand, there was no significant change in duration of D features in poor performers, even when performing the mental arithmetic task (Fig. 2; left columns). There was a difference in the occurrence of type D feature, depending on performance level in the task state (Fig. 2; right columns). Across type features, the mean duration decreased and the mean occurrence increased during the task state in both groups. However, during the task, poor performers showed greater change than good performers. In other words, good performers maintained temporal stability better than poor performers (Fig. 3; left and middle columns). Therefore, mean duration and mean occurrence during the task (right Fig. 3; right columns) differed between the two groups. These data suggest that type D features, mean duration, and mean occurrence are suitable to reflect mental arithmetic task performance.

### Transition probability

**Figure 4:**
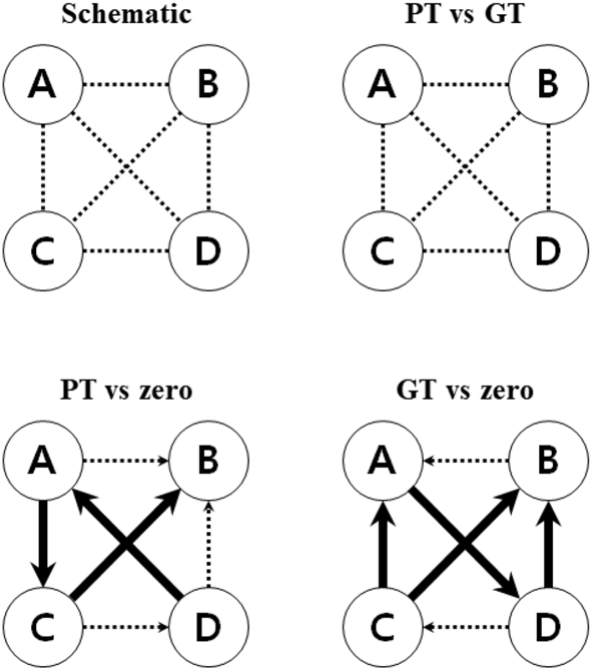
Directional predominance of microstate sequence during mental arithmetic task. The letters in the circle represent the microstate of each type. Solid arrows indicate that the transition from one microstate to another is significantly increased compared to the opposite direction between two groups after Bonferroni–Holm correction. The dotted line indicates that there is no statistical significance, and the direction is indicated by arrows in comparison with zero. *PT* poor performers in task state, *GT* good performers in task state.

The transition probabilities in the four states are presented in Table 2. During the task performance, there was no significant difference between poor performers and good performers in the 11 possible transitions except (A → D) (p = 0.034). For directional predominance, it was analysed whether the mean values of poor and good performers deviated from zero. In poor performers, (A → C) − (C → A) increased (p = 0.009) (A → D) − (D → A) (p = 0.001) and (B → C) − (C → B) (p = 0.002) decreased. Accordingly, the dominant microstate sequence was D → A → C → B. In good performers, however, (A → D) − (D → A) (p < 0.001) increased, while (A → D) − (D → A) (p < 0.001), (B → C) − (C → B) (p < 0.001), (B → D) − (D → B) (p < 0.001) decreased. Thus, the dominant microstate sequence of good performers was C → A → D → B. In other words, when performing a task from the resting state, there was a difference in the dominant microstate transition according to task achievement. However, there was no difference in the directional predominance between resting and task state in poor performers while performing the mental arithmetic task (Fig. 4). There was also no difference in transition probabilities between good performers and poor performers during task performance.

**Table 2:** Twelve transition percentages and six directional predominance features were obtained from good and poor performers during task performance.

A. Transition percentages (from a class → other class)												
	A → B	B → A	A → C	C → A	A → D	D → A	B → C	C → B	B → D	D → B	C → D	D → C
**PT**												
Mean (sd)	4.08 (4.06)	4.06 (4.09)	8.11 (5.20)	7.27 (4.96)	6.03 (4.85)	6.88 (4.96)	8.10 (6.44)	9.00 (6.02)	8.03 (5.46)	7.44 (5.40)	13.57 (6.71)	13.31 (6.43)
**GT**												
Mean (sd)	4.04 (4.09)	4.05 (3.97)	7.31 (5.32)	7.43 (5.58)	6.25 (5.08)	6.14 (5.04)	9.08 (6.33)	9.11 (6.25)	7.93 (5.80)	8.00 (5.63)	13.07 (6.80)	13.28 (6.97)
Difference	0.04	0.01	0.80	− 0.16	− 0.22	0.74	− 0.98	− 0.11	0.10	− 0.56	0.50	0.03
z-value	0.165	2.313	− 0.565	− 0.361	− 2.385	0.378	− 0.001	− 0.147	0.949	2.314	− 1.428	0.072
Adjusted p -value	> 0.999	0.083	> 0.999	> 0.999	0.034*	0.706	> 0.999	0.938	0. 685	0.062	0.460	0.943

B. Directional predominance												
	A → B − B → A	A → C − C → A	A → D − D → A	B → C − C → B	B → D − D → B	C → D − D → C						
**PT**												
Mean (sd)	0.02 (4.74)	0.84 (5.46)	− 0.85 (5.19)	− 0.90 (5.84)	0.60 (5.76)	0.26 (6.28)						
p-value (vs zero)	0.951	0.009*	0.001*	0.002*	0.181	0.952						
**GT**												
Mean (sd)	− 0.01 (4.31)	− 0.12 (5.45)	0.10 (5.26)	− 0.04 (6.11)	− 0.06 (5.98)	− 0.20 (6.70)						
p-value (vs zero)	0.848	< 0.001*	< 0.001*	< 0.001*	< 0.001*	0.816						
Difference	0.03	0.96	− 0.95	− 0.86	0.66	0.47						
z-value	− 0.036	2.302	− 2.383	− 2.284	1.570	0.978						
Adjusted p -value	> 0.999	0.085	0.069	0.067	0.233	0.985						

Statistical analyses were performed using Mann–Whitney test. After performing Bonferroni–Holm correction for multiple comparisons, significant differences are indicated in asterisk and bold.

*PT* poor performers in task state, *GT* good performers in task state, mean (standard deviation).

### Feature selection using recursive feature elimination and classification results

**Figure 5:**
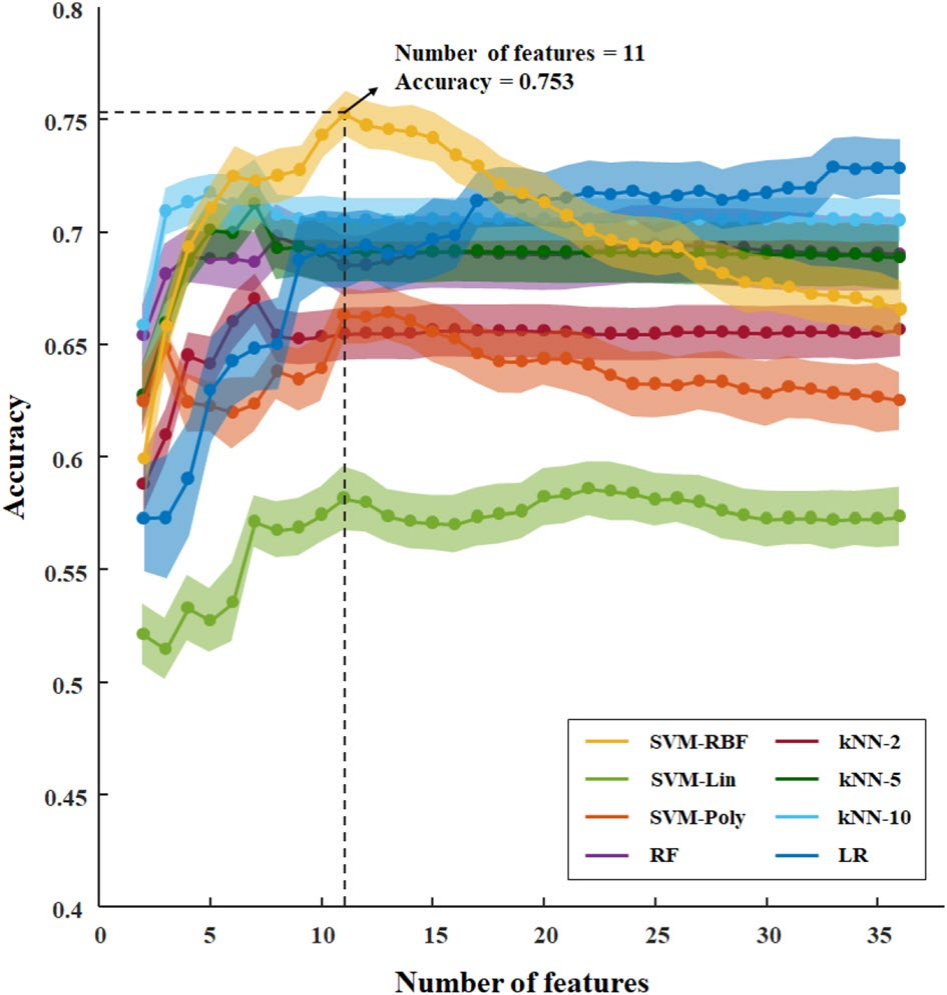
A recursive feature elimination step with different classifiers to select optimal number of microstate features that best differentiate poor performers and good performers. Mean accuracy is presented as a percentage. The shaded area represents the variability of 100 times permutation, one standard deviation above and below the mean accuracy shown by the point. Maximum mean accuracy, 75.3%, is obtained using RBF-kernel SVM classifier to select 11 features from the original feature set of 36 features. *RFE* recursive feature elimination, *SVM* support vector machine, *Lin* linear kernel, *Poly* polynomial kernel, *RBF* radial basis function, *kNN-n* k-nearest neighbours with k value of n, *RF* random forest, *LR* logistic regression.

RFE was implemented to determine the features that best distinguish the two groups with different task performance. The feature selection step using several classifiers is shown in Fig. 5. Based on the classification accuracy, the best result of 75.3% was obtained with RBF-kernel SVM classifier when the number of selected features was 11. The selected features are as follows: mean occurrence, four (A, B, C, and D) occurrences and four GFPs, coverage of type C and D microstates.

**Figure 6:**
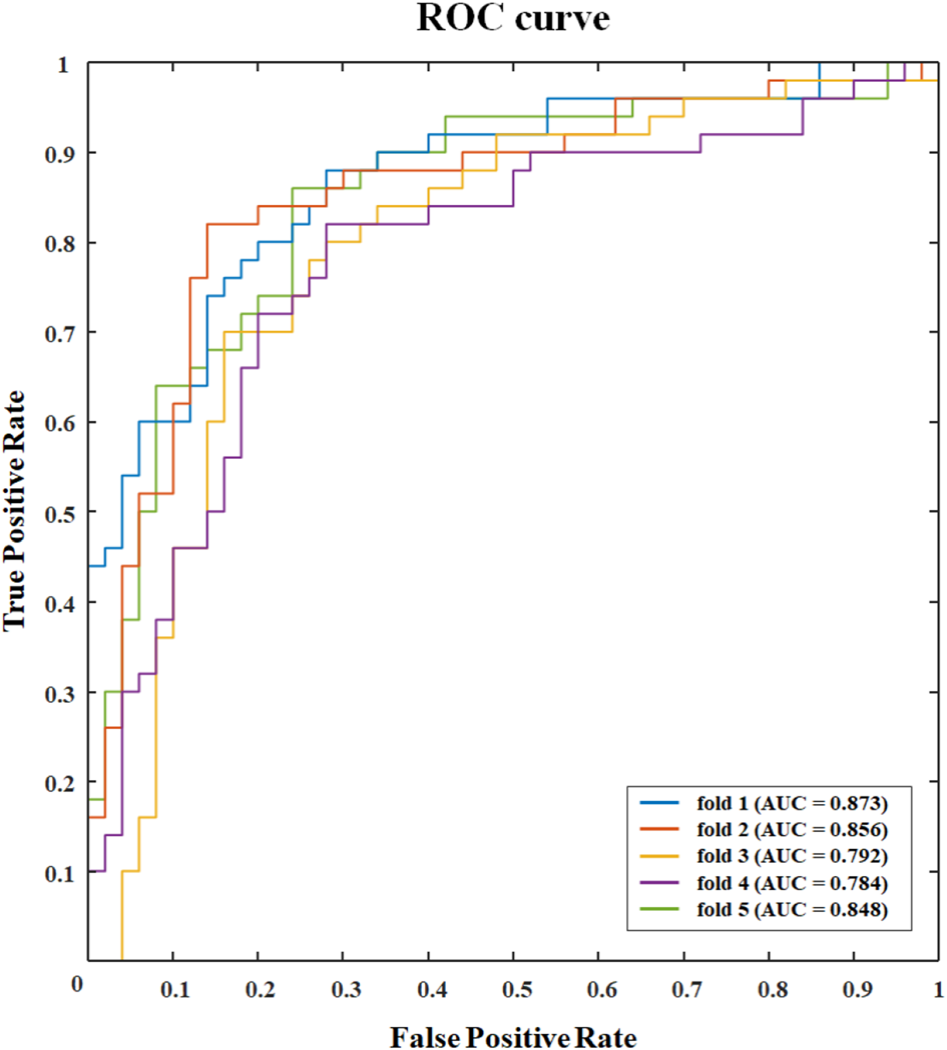
ROC curves for each fold in the classification using RBF-kernel SVM classifier with eleven microstate features selected by recursive feature elimination show a mean AUC of 0.831.The AUCs for each fold are as follows: 0.873, 0.856, 0.792, 0.784, and 0.848. *ROC* receiver operating characteristics, *RBF* radial basis function, *SVM* support vector machine, *RFE* recursive feature elimination, *AUC* area under the curve.

Table 3 shows the comparison of results that differentiate between good performers and poor performers using several classifiers with 11 features selected by RFE. The mean accuracy, AUC, sensitivity, and specificity were calculated by five-fold cross-validation. In mental arithmetic task performance classification, the highest mean AUC of using eleven features selected by feature selection was 0.831. The ROC curves for each fold of the RBF-kernel SVM classifier, which showed the highest performance, are illustrated in Fig. 6.

**Table 3:** Classification performance achieved using eleven microstate features selected by recursive feature elimination from task state EEG recordings in good and poor performers.

Microstate features	LR	SVM-Lin	SVM-Poly	SVM-RBF	kNN-2	kNN-5	kNN-10	RF
**Poor vs good**								
Accuracy (%)	0.702	0.590	0.664	**0.764**	0.664	0.732	0.720	0.706
AUC	0.755	0.610	0.718	**0.831**	0.664	0.763	0.784	0.761
Sensitivity (%)	0.717	0.592	0.664	**0.767**	0.669	0.719	0.690	0.694
Specificity (%)	0.694	0.590	0.674	**0.767**	0.667	0.750	0.768	0.744

The results were obtained from five-fold cross validation. The highest classifier performance is bold.

*LR* logistic regression, *SVM* support vector machine, *Lin* linear kernel, *Poly* polynomial kernel, *RBF* radial basis function, *kNN-n* k-nearest neighbours with k value of n, *RF* random forest.

## Discussion

Using EEG microstate analysis, we examined whether task performance was reflected in microstate features. First, we divided subjects into good performers and poor performers based on how well they performed on a mental arithmetic task. Second, we investigated how microstate features changed in good performers and poor performers when conducting the task compared to resting state. Next, we compared the microstate features of good performers and poor performers during the task state. Finally, we evaluated which of the microstate features is best at assessing task performance using several classifiers.

Most of our results are in line with the hypothesis that performance on mental arithmetic tasks is reflected in microstate features. In the task state of the poor and good performers, most type A and B features associated with the sensory network did not change, type C features associated with the default mode decreased, and type D features associated with dorsal attention increased compared to the resting state. Considering that a mental arithmetic task is one requiring attention, working memory, and executive functions, this result supports the previously accepted functional significance^25,26^. This is also consistent with the results of Seitzman et al. showing that microstate type D is task-positive, and microstate type C is task-negative^27^. In both groups, the mean duration and mean occurrence changed, which suggests that there was a change in temporal stability due to task execution^28^. However, poor performers had a different degree of change compared to good performers. Type D features increased less and the change in mean duration and mean coverage was greater. While there was no difference in type C features during the task and the changes in duration and coverage by the task were similar, occurrence of type C feature decreased only in poor performers. As a result, poor performers showed reduced type D features, increased mean duration, and decreased mean occurrence compared to good performers during the mental arithmetic task. In particular, task-positive type D features, which are known to be related to dorsal attention, differed in occurrence.

These results support previous fMRI findings on cognitive function and microstate analysis. Depending on the required cognitive function, higher-order brain tasks require flexible coupled activity of the default mode network and dorsal attention network^42^. In order to perform the mental arithmetic test, the subject must encode the presented information, perform the calculation, provide the response, and maintain the intermediate result^43^. This task is suitable for our study because it can minimise noise caused by eye movements, and is relatively less involved in other sensory systems. Moreover, it was a multidigit subtraction arithmetic task, which is more difficult than the normal level, requiring considerable attention. It is a goal-directed (top-down control) task that is associated with the three working memory systems, activating the dorsal attention system^44^.

Britz et al. have reported that type D and type C microstates are associated with the dorsal attention system and default mode network, respectively^25^. Type D microstate features increased during the task in the study using serial subtraction tasks^27^. Considering the association between the type D microstate feature and the dorsal attention system, the mental arithmetic task requiring the attention system would have resulted in an increase in the type D microstate features. In addition, our results suggest that type D features can reflect not only task execution but also task achievement. The activation of the dorsal attention system leads to good performance, which may be reflected in type D features. In contrast to type D features, type C microstate features are known to decrease in tasks that require externally directed cognition^45^. Type C microstate features reflect the default mode network^25,27^, which is a large-scale distributed network that shows task-induced deactivation^46,47^. Similar to several fMRI studies, task-positive dorsal attachment network and task-negative default mode network are antagonistic^48,49^, and type C and D microstate features have been reported to show antagonistic inclination in both healthy subjects^24,27^ and disease groups^40,50,51^. The deactivation of the default mode network improves the performance during goal-directed tasks, reflected in type C features. Taken together, the antagonistic activation of the dorsal attention network and the default mode network is required to perform the mental arithmetic task better, and this may lead to an increase in the type D microstate feature and a decrease in the type C microstate feature.

The transition probability of EEG microstate analysis has been applied in sleep^33^, schizophrenia^30^, mood, and anxiety^52^ studies. The results of previous studies showed that impairment in temporal organisation represents altered transition probability^30,53^. In our study, the predominant directions deviated from the zero value in both groups during the task state. D → A → C increased in poor performers, while in good performers, C → A → D increased; thus, the increase or decrease in C was opposite in both groups. However, there was no difference in the transition probabilities except for (A → D) between good performers and poor performers during task states. In other words, several microstate features in the two groups were different during the task, but transitions between each microstate were maintained, regardless of achievement of the task. The subjects of this study were healthy subjects who did not have impairment in cognitive functions of the present and past. Thus, the difference in cognitive function of this subject group is much less than that of previous studies. Taken together, we concluded that transition probability can reflect a considerable change in cognition, but is not suitable for reflecting the degree of difference in task achievement.

The results of the several classifiers for classification revealed how well microstate features evaluated task performance, and identified the optimal feature set. Using recursive feature elimination, we selected eleven of the 36 microstate features that most effectively distinguished the two groups. The 11 features included all four archetypes microstate features, and coverage was included in the case of type C and type D. Although type A, B features and three GFPs did not differ significantly between the two groups during task performance, they were useful to obtain higher accuracy as informative features with machine-learning-based multivariate analysis. The highest AUC 0.831, which is generally considered at the level of excellent discrimination^54,55^. Given that they are only microstate features from a two-second epoch, it would be expected to show higher accuracy when applied with other features. These EEG microstate features not only showed characteristic changes depending on task achievement, but also proved most useful for evaluating task achievement. This suggests that it may be possible to objectively evaluate task achievement using these EEG microstate features.

In particular, mean occurrence and type D features were selected by RFE as well as significant differences during the task. As mentioned, type D features and mean occurrence are theoretically meaningful features known to be related to the dorsal attention system^25–27^ and temporal stability of and network^28^, respectively. First, microstate features in our study were obtained from the archetype microstate. Our approach makes it possible to obtain microstate features from those of the same prototype even in different tasks or diseases, so we can compare and interpret the results based on previous studies using the same method. Next, the reliability and validity can be improved by exploiting multiple features simultaneously using multivariate analysis with machine learning classifier. The highest AUC of this study is not only an excellent level of discrimination, but also based on meaningful features based on previous studies. Considering that EEG microstate analysis has information that other modalities cannot provide, our selected features can help to obtain higher performance with features of other modalities. Thus, knowledge-based features obtained from archetype microstate features and selected as theory-driven features can be applied to evaluate cognitive performance.

The mental arithmetic task is easy to administer, minimises activation of other systems, and involves attention, working memory, and executive functions, which are related to general intelligence^56,57^. Mean duration, mean occurrence, and type D features differed depending on the level of performance on this task. This indicates that the dorsal attention system is activated while maintaining temporal stability and usage. EEG microstate features can exhibit these appropriate alterations and have the potential to be used for task achievement evaluation. To our knowledge, this study is the first to evaluate cognitive task performance in healthy subjects using EEG microstate features. Human cognitive function is related to the spatiotemporal dynamics of brine, as determined by various neuroimaging methods^58,59^, especially fMRI^60^. However, since the BOLD response in fMRI has a delay of several seconds and is affected by local vasculature^2^, it is impossible to perform fine temporal analysis of brain activity^60^. A method that permits the measurement of brain dynamics should be selected for proper evaluation of cognitive function, considering the time course of neural activity. EEG has excellent temporal resolution, sufficient to analyse components of event-related potentials. In other words, microstate analysis can exploit the advantages of EEG to investigate sub-second brain dynamics required for human cognitive process research^61–63^. Previous work has shown the EEG microstate features can reflect several diseases; our results show that they can also reflect differences in the cognitive function of healthy subjects. This approach is expected to help objective measurement of intelligence and provide a deeper understanding of brain function.

Some limitations of this study should be discussed. This work is based on data from a public dataset consisting of a small number of subjects. Therefore, there is a limit to the generalisation of these results. Large cohort studies that can represent the general population are needed in the future. Next, among several EEG microstate analysis methods, the k-means clustering method was selected and the k value was fixed at 4. Better explained variance can be obtained with different clustering methods^64^ or using different k values^27^. However, the k-means clustering method with a k value of 4 has its own advantages. According to the cross-validation criterion introduced by Pascual-Marqui et al.^64,65^, the k value that maximises the explained variance was presented as 4. The method was valid to show the variance on the average of 79% in the resting state EEG in a large control group of 496 people. In the study conducted by Seitzman during tasks^27^, the four archetype microstates showed more than 60% explained variance. It has already been applied in many independent studies^15^, and the function of each archetype is well known^25,26^. After further studies have demonstrated the functional significance of EEG microstates for objective cognitive function evaluation, other clustering methods can be applied to assess cognitive functions more accurately using similar methods. In summary, given that few studies have applied EEG microstate analysis to specific tasks in healthy subjects, our results are valuable by demonstrating that generalisation of this approach appears promising.

## Conclusions

In this study, we demonstrate the usefulness of EEG microstate features in evaluating cognitive performance. Our results show that EEG microstate features differ depending on performance on a mental arithmetic task. In particular, type D features increase, type C features decrease, mean duration increases, and mean occurrence decreases in good performers. This is because the EEG microstate features may reflect the activation of the dorsal attention system, deactivation of the default mode network, and ability to maintain temporal stability. The features selected by RFE include these meaningful features, and they showed an excellent level of discrimination in distinguishing good performers from poor performers. This suggests that EEG microstate features can be helpful in the objective evaluation of cognitive function. Further studies are needed to assess the value of EEG microstates in other tasks requiring different cognitive functions, and in the general population.

## Methods

### Dataset

A publicly accessible EEG dataset was analysed in this study, which was obtained from the ‘EEG During Mental Arithmetic Tasks’^66^ in the EEG dataset on PhysioNet.org^67^, was approved by the Bioethics Commission of Educational and Scientific Centre ‘‘Institute of Biology and Medicine,’’ Taras Shevchenko National University of Kyiv and written informed consent was obtained from each subject in accordance with the World Medical Association (WMA) Declaration of Helsinki. Healthy university student volunteers with no visual, cognitive, or mental impairment were eligible to participate in the task. The following exclusion criteria were applied: use of psychoactive medication, drug or alcohol addiction, and psychiatric or neurological complaints. Of the 66 initial participants, participants with poor EEG quality were excluded due to eye and muscle movement (based on visual inspection by an expert). A total of 36 subjects were included in analyses, ranging between the ages of 17–26 (9 males, 27 females; 18.25 ± 4.5 years). Information on raw dataset including EEG recording environment and data selection can be found in Zyma et al.^68^.

EEG was recorded at 500 Hz sampling frequency with a 0.5–60 Hz band-pass filter using a Neurocom monopolar EEG 23-channel system (Ukraine, XAI-MEDICA). Silver/silver chloride electrodes were placed in accordance with the International 10–20 System: frontal (Fp1, Fp2), frontal (F3, F4, Fz, F7, F8), central (C3, C4, Cz) parietal (P3, P4, Pz), occipital (O1, O2), and temporal (T3, T4, T5, T6).

### Experimental protocol

**Figure 7:**
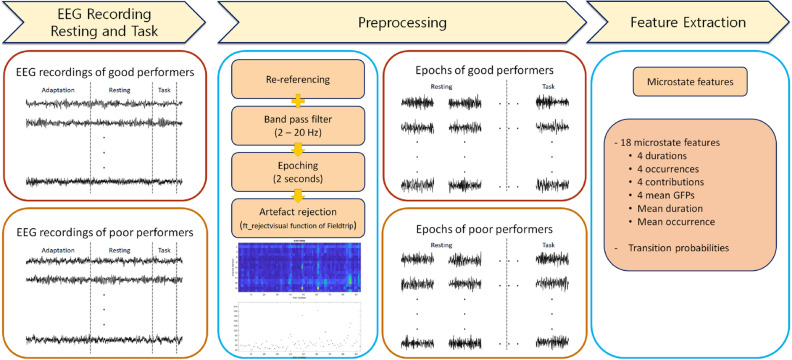
Overview of the process for extracting EEG microstate features. *EEG* electroencephalography, *GFP* global field potential, *PR* poor performers in resting state, *PT* poor performers in task state, *GR* good performers in resting state, *GT* good performers in task state.

A summary of the experimental protocol and preprocessing steps are summarised in Fig. 7. The EEG recordings were collected during the eyes closed resting state and mental arithmetic task state. The task requires activation of the dorsal attention system^69,70^, which is known to be associated with voluntary attentional control^71,72^. During the resting state, after 3 min of adaptation, subjects were asked to relax with their eyes closed. During a mental arithmetic task for 15 min, subjects performed a serial subtraction wherein two digests (subtrahend) were subtracted from four digits (minuend). The subjects were asked to calculate mentally without speech or finger movement. In this study, the 3 min resting state and the first 1 min of the task state were selected for analysis, due to mental fatigue caused by the task later on. The achievement of the mental arithmetic task was assessed according to Zyma et al.^68^ and Seleznov et al.^66^ based on the number of operations performed per minute^73^. As a result, the subjects were divided into 26 good performers and ten poor performers. Finally, four states of EEG recording were analysed: good and poor performers, in the resting state and task state. A detailed description of the experimental design and group selection can be found in Zyma et al.^68^.

### EEG data preprocessing

We implemented a preprocessing step similar to methods described by Damborská et al.^22^ and Schiller et al.^28^, which analysed all data points. Two raw EEGs randomly selected from each group are shown in Supplementary Fig. S1. Preprocessing, microstate analysis and feature extraction were performed by referring to the well-established methods of Koenig et al.^12,24^ and Seitzman et al.^27^. Re-referencing to common average electrode and 2–20-Hz band pass filter were performed according to Koenig's method for deriving four archetypes. For classification using microstate features, continuous raw EEG was segmented in two seconds to enrol more samples. Epochs containing artefacts were removed using the ft_rejectvisual function of Fieldtrip, which shows the variance and maximum absolute value of each channel and allows the visual rejection. Criteria for rejection was that maximum absolute value exceeded 100 μV or variance exceeded 500. The epoch that was not rejected by criteria was subsequently removed by visual inspection, even though it contained obvious artefact. A total of 3704 epochs were obtained from EEG recordings of all subjects. All EEG analysis was implemented using the FieldTrip toolbox^74^.

### Microstate analysis

EEG microstates were analysed as described by Koenig et al.^12,24,28^, one of the most widely used methods. With this method, we obtained four archetype microstates with functional significance found in fMRI studies using the EEGlab toolbox. This measures global electrical activity in instant of time. First, GFP was derived from EEG recordings as follows:

1 $$GFP = \sqrt {{{\left( {\sum\limits_{i}^{N} {\left( {V_{i} \left( t \right) - V_{{mean}} \left( t \right)} \right)^{2} } } \right)} \mathord{\left/ {\vphantom {{\left( {\sum\limits_{i}^{N} {\left( {V_{i} \left( t \right) - V_{{mean}} \left( t \right)} \right)^{2} } } \right)} N}} \right. \kern-\nulldelimiterspace} N}} .$$

where ${V}_{i}\left(t\right)$ and ${V}_{mean}(t)$ denote the instantaneous and mean potentials across the *N* electrodes at time *t*.

Then, successive microstates, which are discrete states of the EEG analysed based on local maxima of the GFP, were derived. Using modified k-means clustering, all microstates were assigned to four archetype microstates as follows: left–right orientation (type A), right-left orientation (type B), anterior–posterior orientation (type C), and a fronto-central maximum (type D)^12,24^.

Once the number of microstates was identified (four archetype microstates), we have to label them into a sequence by using modified K-means clustering algorithm and Global Explain Variance (GEV) criteria^75^. The setting parameters for K-means algorithms are re-run and iterations as explained following. In principle, by re-running the stochastic k-means algorithm multiple times (in this analysis, we set re-run parameter to 20 times), we are able to test multiple segmentations on the same dataset and select the best re-run based on the GEV criteria^75^. GEV is a measure of how similar each EEG sample is to the microstate prototype it has been assigned to. The higher the GEV the better^64^. More importantly, we are able to reach the global minimum among 20 local minimums (20 re-runs). After 20 re-runs, the one that maximises the GEV is selected. However, the number of re-run is a trade-off between computation time and how likely we are to converge on the same optimal solution. In the Microstate EEGlab toolbox^64^ and tis Python package^76^ in which we have applied for our analysis select 10 re-run as a default value. In addition, Thomas Koenig's manual^77^ has recommended that a range from 20 to 50 re-runs could be sufficient for a proper analysis. Furthermore, we have found that there are several existing EEG microstate analysis literatures that set 10 re-runs^65,78^ as well as papers that use 30^20^ as a proper re-run number.

Another parameter for K-means clustering is iteration, which means that in each re-run the K-means algorithm keeps iterating until some stopping criteria (convergence threshold) are satisfied. In this analysis, we used the convergence threshold, which stops the algorithms when the relative error change between subsequence iterations is below the threshold. Here, we set the threshold at 10-6. The maximum number of iterations set to 1000 which means the algorithm can stop if the maximum iteration is reached before convergence for computation time efficiency.

Microstate features were then extracted. In each microstate, duration was defined as the average duration of microstates per second. Occurrence was defined as the average frequency of microstates observed. Coverage was defined as the percentage of each microstate appearing in each epoch. Mean GFP was defined as the average GFP for a microstate. Next, two features were extracted for epochs across all types: mean duration was defined as the average duration of all types in a specific epoch and mean occurrence was defined as the frequency of all microstates per second in each epoch. In summary, a total of 18 microstate features were used in this study: four features for each type and two across all types. Transition probabilities, the percentage of transition from one to the different microstates, were also calculated. Thus, there were a total of 12 pairs. The directional predominance introduced by Lehmann et al. were also analyzed. It reveals the asymmetries of transition between two types of microstates in their sequences. Using the permutation test, the difference between the expected transition and the observed transition was calculated. This process was run 10,000 times to obtain the p-value.

### Feature selection and classification

To examine which of the 36 microstate features is more suitable for performance evaluation, we used several machine-learning algorithms and feature selection methods. Feature selection was performed by selecting the most informative features to help enhance the classification performance and reduce complex computations and overfitting problems. RFE is a multivariate wrapper-based feature selection algorithm that ranks features according to their effects on classification^79^. In each RFE iteration, the lowest-ranking features are removed, whereas the remaining features are used for the next iteration. This sequential process is repeated until all features are discarded and the optimal number of features is determined. Our initial dataset is unbalanced, therefore, we have implemented downsampling strategy, which randomly select a subset of balanced samples from each group. In particular, we selected 250 epochs for each group and used five-fold cross-validation to assess classification performance. That is, for each fold, 200 epochs and 50 epochs in each group of PP and GP are used for training and testing set, respectively. The determination in optimal number of features using RFE was evaluated by repeating 100 times to avoid bias due to random selection. The classifier is selected based on stability and simplicity including both linear and non-linear one; logistic regression (LR), support vector machine (SVM), k-nearest neighbor (kNN), and random forest (RF). The accuracy, AUC, sensitivity, and specificity were derived to compare the classification results.

### Statistical analysis

Because all features obtained from EEG do not satisfy normality, Mann–Whitney test was used to compare the 18 microstate features and transitional probabilities between the two groups. To avoid type I error caused by the multiple-comparison problem, statistical significance was evaluated by applying Bonferroni-Holm correction (corrected p-value < 0.05). All analyses, including EEG preprocessing, were performed with MATLAB software.

## Supplementary Information

Supplementary information.

## Data Availability

All datasets presented in this study are openly available in PhysioNet at https://doi.org/10.13026/C2JQ1P. All epochs that have been preprocessed are vailable on Figshare at https://doi.org/10.6084/m9.figshare.13135130.
