# Supplementary material for: EEG microstate features according to performance on a mental arithmetic task
Source: Sci Rep. 2021 Jan 11;11:343. doi: 10.1038/s41598-020-79423-7 (PMC7801706; doi:10.1038/s41598-020-79423-7)

Supplementary files

Title: EEG Microstate Features according to Performance on a Mental Arithmetic Task

Authors: Kyungwon Kim, Nguyen Thanh Duc, Min Choi, Boreom Lee*

Author's Information

Kyungwon Kim, M.D.

Department of Biomedical Science and Engineering (BMSE), Institute Integrated Technology(IIT), Gwangju Institute of Science and Technology (GIST), 123 Cheomdan-gwagiro, Buk-gu, Gwangju, Korea. 61005.

E-mail address: kyungwonkim@gist.ac.kr

Nguyen Thanh Duc, Ph.D

Department of Biomedical Science and Engineering (BMSE), Institute Integrated Technology(IIT), Gwangju Institute of Science and Technology (GIST), 123 Cheomdan-gwagiro, Buk-gu, Gwangju, Korea. 61005.

E-mail address: ducnguyen@gist.ac.kr

Min Choi, M.S.

Department of Biomedical Science and Engineering (BMSE), Institute Integrated Technology(IIT), Gwangju Institute of Science and Technology (GIST), 123 Cheomdan-gwagiro, Buk-gu, Gwangju, Korea. 61005.

E-mail address: minchoi1021@gist.ac.kr

Corresponding author: Boreom Lee, M.D., Ph.D.

Department of Biomedical Science and Engineering (BMSE), Institute Integrated Technology(IIT), Gwangju Institute of Science and Technology (GIST), 123 Cheomdan-gwagiro, Buk-gu, Gwangju, Korea. 61005.

Tel.: +82-62-715-3272; Fax: +82-62-715-3244

E-mail address: leebr@gist.ac.kr

Supplementary files

Supplementary Figure S1. Raw EEG during resting state of two participants in randomly selected groups among poor and good performers.

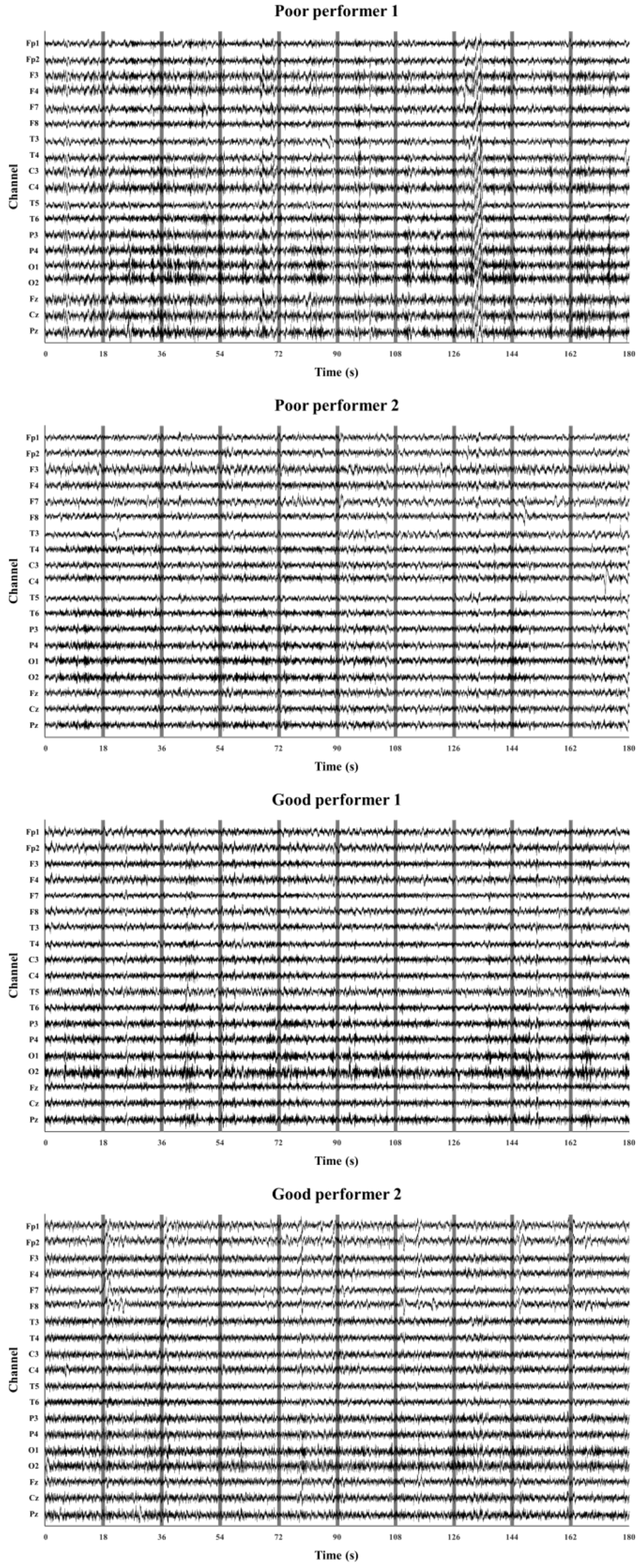

Supplement: Supplementary file 1 — Supplementary information. [file 41598_2020_79423_MOESM1_ESM.pdf]
